# Orientation-Induced Effects of Water Harvesting on Humps-on-Strings of Bioinspired Fibers

**DOI:** 10.1038/srep19978

**Published:** 2016-01-27

**Authors:** Yuan Chen, Dan Li, Ting Wang, Yongmei Zheng

**Affiliations:** 1 Key Laboratory of Bio-Inspired Smart Interfacial Science and Technology of Ministry of Education, School of Chemistry and Environment, Beihang University, Beijing, 100191 (P. R. China).

## Abstract

Smart water-collecting functions are naturally endowed on biological surfaces with unique wettable microstructures, e.g., beetle back with “alternate hydrophobic, hydrophilic micro-regions”, and spider silk with wet-rebuilt “spindle-knot, joint” structures. Enlightened by the creature features, design of bio-inspired surfaces becomes the active issue in need of human beings for fresh water resource. Recently, as observed from spider web in nature, the net of spider silk is usually set in different situations and slopes in air, thus spider silks can be placed in all kinds of orientations as capturing water. Here, we show the styles and orientations of hump-on-string to control the ability of water collection as bioinspired silks are fabricated successfully. As different strings, sizes (height, length, pitch) of humps can become the controlling on volumes of extreme water drops. It is related to the different solid/liquid contact regions resulting in the as-modulated wet adhesion due to orientations of humps-on-strings. The conversion of high-low adhesion can be achieved to rely on orientations for the effect of capturing water drops. These studies offer an insight into enhancement of water collection efficiency and are helpful to design smart materials for controlled water drop capture and release via conversions of high-low adhesion.

Bioinspired micro-/nano materials with special functions have extensive influence on material sciences. Biological unique and excellent microstructure surfaces have developed a great promising field of functional materials on fabrication and design. The most noteworthy creatures show unique wettability properties on their surfaces^1^,^2^,^3^,^4^,^5^,^6^,^7^, e.g., back of desert beetle^1^, spider silk^2^, and cactus spines in the desert^7^. The cribellate spider takes advantage of the “periodic bead-on-string” structures of silk to collect water from fog^8^. The wettability^9^,^10^ and structure gradient^11^,^12^,^13^,^14^,^15^ on the wetted capture spider silks are cooperated to move tiny water droplets. Numerous methods such as dip-coating or multiple dip-coating^7^,^16^,^17^, fluid coating^18^, uniaxial or coaxial electro-spinning^19^,^20^, microfluid^21^ and Rayleigh-Plateau instability^22^,^23^ are developed to fabricate the bioinspired fibers from micro-level to nano-level. Thus water collection accompanying with driving of tiny droplets is achieved on bioinspired fibers that can be also regulated by roughness^24^,^25^, light or thermal responses^26^,^27^. Observed from spider web in nature, the net of spider silk is usually set by different situations and slope in air for capturing water, where spider silks can be carried out in all kinds of orientations. However, it is quite unclear how it is orientations of silks to be related to the adhesion of interfaces for water capture in efficiency so far.

Inspired by the features of orientations in silk net, here, we show the styles and orientations of hump-on-string to control the ability of water collection as bioinspired silks are fabricated successfully. We reveal effect of the sizes (height, length, pitch) on humps-on-strings for the controlling on the volumes of extreme water drops, and elucidate the relationship between the solid/liquid contact regions, wet adhesion and orientations of humps-on-strings on as-fabricated fibers, and finally find out a way that the conversion of high-low adhesion is relied on orientations of humps-on-strings for the efficiency of capturing drops. These studies offer an insight into enhancement of water collection efficiency and are helpful to design smart materials for controlled water drop capture and release via conversions of high-low adhesion.

The sizes of humps-on-strings can be a key to enhance water collection in efficiency. Some researchers have been discussed the effect of drawing velocity and concentration of polymer solution on the size of humps^17^. Recently, it is found that the diameters of main fibers can influence the formation of humps in sizes after preparation via Rayleigh instability technique^28^ and the dip-coating method. An original uniform nylon fiber is dipped in the PVDF/DMF solution with a drawing out velocity. A polymer solution film would be uniformly coated on this fiber. Then the film is quickly broken up into multi-level humps via Rayleigh instability. In our study, we use the main fibers with different diameters (e.g., d = 20 μm, 45 μm, 80 μm, and 98 μm, where d is diameter of fiber) to be immersed into poly(vinylidene fluoride, PVDF, M_w_ = 530000)/DMF (N,N-dimethylformamide, 9%, 10%, 11% or 12% (wt)) solutions. After being drawn out of the PVDF/DMF solution with a velocity 200 mm/min, the as-coated polymer solution film is quickly broken up into periodic droplets. Dried after 10 min in the 60 °C, the humps-on-strings form on fibers. Figure 1a shows the optic images of a series of bioinspired fibers with humps-on-strings. The diameters of strings are near to diameter of fiber due to formation of periodic polymer droplets by Rayleigh instability. As for the fabrication of bioinspired fiber^9^,^17^, the polymer solution is one of crucial importance influence factor, e.g., given fiber (d = 20 μm), the size (e.g., L, H) of humps with higher concentration of solution (10% and 11%) are bigger than that with lower one (9%). This is because there is more PVDF solution over on the fiber after drawing out the solution with the higher concentration. But it doesn’t mean that the size of hump is directly proportional with solution concentration. For an excessive high concentration, it would generate a high gravity to deform the formation of humps. The red sphere between the dotted lines in Fig. 1a has vividly shown that the parameters present a parabola with the concentration increasing. Figure 1b illustrates the statistical data of solid/liquid contact surface as fabricated under different conditions including changeable solution concentrations on main fibers (e.g., d = 45 μm, 80 μm, and 98 μm, respectively). The solid/liquid contact areas are calculated by 
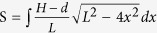
, where H is the semiminor axis of hump (the height of hump); L is the semimajor axis long the surface of normal fiber and d is the diameter of string (estimated by diameter of main fiber); x is the one side of solid-liquid contact line along axis of fiber; dx is the integrating unit line of solid-liquid contact at x position. Thus the solid-liquid contact areas for larger hanging-droplets can be predicted by optimizing the concentration of polymer and diameter of main fiber. As for diameters for main fibers with d = 20 μm, 45 μm, 80 μm and 98 μm, the proper concentration can be 10%, 10%, 11% and 12% for excellent formation of humps, respectively.

As reported^15^, the hanging water droplet ability is related to the three-phase contact line, which is in proportion to the adhesion force at solid-liquid interface. Figure 1c shows the photos of droplets that are hung on humps of fibers (i.e., d = 20 μm and 98 μm), where the volume of water droplet can be expressed with 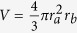. It indicates that larger hump increases the solid-liquid contact area to hang the larger hanging-droplet effectively. Figure 1d shows the relationship between volumes of hanging-drops and solid-liquid contact areas. The statistical data of the solid/liquid contact area of one hump versus hanging-drop volume is fitted by the testing data. Given the same 10% PVDF solution coated on different main fibers (i.e., d = 20 μm, 45 μm, 80 μm and 98 μm), the solid-liquid contact area can be different for volumes of hanging-droplet. The volumes of water droplets are increasing with the solid/liquid contact area increasing. The solid-liquid contact areas are determined by geometries of humps formed finally on fiber. We define the curvature of the hump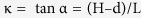, where 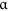 is the semi-axis angle; H is the hump of height; L is the length of hump, 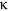 is directly related to the Laplace pressure in difference. Figure S1 shows the 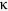 value versus concentration of PVDF (9%, 10%, 11% and 12%) for main fibers with different diameters. The average of 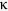 value obviously decreases with the diameter increasing, which means the diameters of main fibers result in the differences of hump geographies, in addition to polymer solution concentrations and drawing velocities. Figure S2 shows the obvious tendency with diameter increasing at 9%. We also prepared different categories of fiber including nylon (d = 20 μm), silkworm silk (d = 23 μm), copper wire (d = 65 μm) and fishing line (d = 163 μm) in Figure S3. The change of 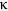 value is induced by materials property and diameter of main fiber. When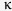 value is bigger, namely, the diameter of main fiber is less than others. The semi-axis angle is too big which results that the water droplet is easy to slip down from the string part to hump surface. The solid/liquid contact area is sharply decreasing, which results that the experimentally area is less than the calculative value. The volume of hanging water droplet is decreasing accordingly. When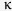 value is too small with a bigger diameter of fiber string, even the solid/liquid contact area is not big enough, the volume of water droplet is more than that bigger solid/liquid contact area humps with thin diameter. It results from the actual extensive contact area on string between humps. It is visible that the topography directly affects the solid/liquid contact area, which influences the water hanging ability^29^. Specifically, environmental SEM images of periodic humps of spider silk with the half apex angle of 9.5° ± 2.5°, κ is 0.172 ± 0.02. For the humps with middle κ value, the relation of water droplet volume and solid/liquid contact area can be depicted by: 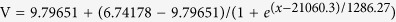, where the volumes of hanging droplets can be evaluated effectively according to the sizes of humps.

Bioinspired fibers weaving net would be expected to have a promising application for water collection, e.g., fog collectors^30^,^31^,^32^. In the process of water collection, droplet is collected usually on multi-humps. Especially, the efficiency of water collection is determined by structures of humps-on-strings on a fiber. The wet adhesion to droplet would be determined by multi-humps. For instance, a droplet is stably hung on two humps with two κ values. Two fibers (i.e., d = 20 μm and 98 μm) have the same pitch of both humps (P = 865 μm), the humps-on-strings are obliquely placed at tilt angles (β = 0°, 5°, 10°, 20°, 30°, 40°) in humidity of ~95%. Figure 2a shows the photos of hanging-drops pinned by humps-on-strings of both fibers (defined as d_20_ fiber and d_98_ fiber). The most volumes of water drops versus the collecting times are both decreasing with the tilted-angles increasing. The collecting time of the d_20_ fiber (the top, d = 20 μm, H = 146 μm, L = 397 μm, 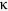 = 0.317) is sharply reducing from ~103.7 s at 0° to ~27.8 s at β = 40°. The d_98_ fiber (the bottom, d = 98 μm, H = 347 μm, L = 1032 μm, 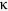 = 0.117) demonstrates the identical changeable tendency. The details can be shown in Figure S4. Water collecting time on the second fiber declines from ~139.2 s to ~49.2 s. The d_98_ fiber with more string diameter presents more drop volume and more collecting time in the once water collecting process. We define efficiency in single-cycle φ = *V*_*max*_/*t*_*max*_, where *V*_*max*_ and *t*_*max*_ are the most volume of water drop and collecting time once water droplet hanging process at the same title angle, respectively. In multiple repeated statistical analysis, not only for the d_20_ fiber with more
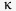
value, but also for the d_98_ one with less 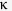
value, both of the two φ values are appearing a peak value between 5° and 10° (Fig. 2b). It implies that a high adhesion state can bear a bigger water drop in single-pass water collecting process in the horizontal situation, but it does not mean the highest collecting efficiency. The adhesion force is adjusted by a title angle. Then, water drop is hanging on a low adhesion state. They can quickly slip down in a transient time. It can promptly circle into next water collecting process. The efficiency achieves the extremum at humps-on-strings with β = 10°. We introduce the circulation collecting water rate with different tilt angle to illustrate the collection efficiency. The cycle-index is stipulated for five times. The water collection efficiency in multi-circles: Ψ=*V*_*total*_*/t*_*total*_, which the *V*_*total*_and *t*_*total*_ are respective the total water collection volume and collecting time during water hanging in multi-cycles. As shown in Fig. 2c, multiple circulation water collecting also is generated an extreme value at β = 10°. Notably, the water drop collecting rate is obvious higher than single-pass process. This results from the coalescence with peripheral tiny water droplet on the string. It follows that the adhesion station decides the most volume of hanging water drops, but the highest collecting efficiency appears on the lower adhesion station. The significance of this work is that we can attempt to use different adhesion station to complete water collection. Based on the efficiency of circulation, suitable adhesion station should be chosen in our application.

As for the water capturing application, we utilize the statistical data to prove that high efficiency water collecting is derived from fleetly water droplet condensation and drop out. Changing the adhesion station not only can improve the water efficiency, but also can release the hanging water droplets. In our research, we further provide four adhesion stations, including horizontal-humps, slope-humps, uniclinal-humps and double-incline-humps, as illustrated in the optical images of d_20_ and d_98_ fiber in Fig. 3a,b. Different adhesion states represent discriminative hanging water drop ability. Here, we depict the most volume of hanging water drops between two humps under four adhesion states. As shown in Fig. 3a, the d_20_ fiber can be H = 120 ± 10 μm, L = 280 ± 20 μm, P = 700 μm. P is pitch between two humps. The red line explicitly reveals the outline changing of solid/liquid contact line under different adhesion states. The most hanging water drop volume also assumes sharply decreasing with the changing from high adhesion to low adhesion state. There are V = 7.73 μL, 6.30 μL, 4.25 μL and 3.15 μL under four adhesion states, respectively. Water drops will release quickly once the volume exceeds the most value. Meanwhile, for the most hanging water drop volume, the humps on d_98_ fiber also show the similar regularity, as demostrated in Fig. 3b. We choose the same P = 700 μm, d = 20 μm, H = 200 ± 10 μm, L = 640 ± 20 μm, respectively. From the optical images, the red solid/liquid contact line shows the same changeable feature. For horizontal-humps, slope-humps, uniclinal-humps and double-incline-humps adhesion states, there are V = 13.11 μL, 10.88 μL, 9.02 μL and 5.74 μL, respectively. However, for the uniclinal-humps and double-incline-humps adhesion states, the volume is the most volume hanging on two humps, but not the most growing volume before dropping down instantaneously. Namely, the drop hanging between two humps is not the most volume. It is different from the thinner bioinspired fiber, e.g., d = 20 μm. As for horizontal-humps and slope-humps adhesion states, the most volume of water drop hangs on two humps. They are dropping along the contact line and slipping out from the humps. But for the uniclinal-humps and double-incline-humps adhesion states, when the droplet drops from the hump to the joint-part, the right end of left hump or the left end of right hump, the water drops are sequentially growing until the adhesion force unable to compete the gravity, which results in the most hanging volume appearing on one hump and uniform fiber string, and not between two humps. This implies that the adhesion state is not only related to the contact area of hump and water drops, but also to the fiber string diameter. Detailed discussion about relationship of adhesion state and hanging water drop volume will be given in the next part.

Figure 3c illustrates the difference of hanging water drops condition on four adhesion situations. The scheme of horizontal-humps (H_h_), slope-humps (S_h_), uniclinal-humps (U_h_) and double-incline-humps (D_h_) shows the change on the apparent receding angle θ, the semi-apex-angle 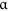 of humps and solid/liquid contact surface (the red dotted line). As known, a large water drop hangs stably on a thin fiber, which is attributed to the solid/liquid adhesion by the wetted feature^28^. Generally, the transformation of high and low adhesion states is decided by adhesion force, which is balanced by the gravity of hanging-drop. However, the adhesion force is provided by two humps and the uniform fiber between them. The capillary adhesion for a larger pearly hanging-drop on a rough-curve fiber can be thereby described as: 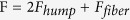, where 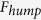 is the combination of the two forces, including the capillary adhesion of knots and the compensatory factor on the difference in Laplace pressure resulting from the geometry of the humps. The description of knots force is introduced as follows: 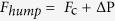, here, the capillary adhesion force is described as 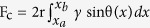, where *x* is the coordinate variable along the fiber axially, 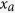 and is the initial sites located at the end of left hump for the integration and 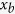 is the terminate one at the right end in one hump, 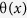 is the crossing angle between the liquid surface and horizontal reference plane, r is the roughness of geometry of bioinspired humps, γ is the surface tension of water. And ΔP can be described as:
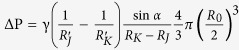
, which is related to the local radius of the hump and string part 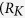 and 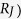, the local curvatures of the contact lines of the drop at the two opposite sides along the fiber 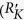 and 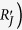, 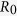 is the radius of water drop and α the semi-apex-angle of hump. For H_h_ and D_h_, the left knot and right knot have the same apparent receding angle θ, and the semi-apex-angle of hump *α*. However, for the S_h_ and U_h_, both θ and α are asymmetrical on the left hump and right hump. Additionally, the solid/liquid contact lines (the red dotted line on the knots) are changing in four conditions. These result in difference of 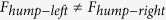 and 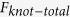 in four state conditions. Another force 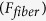 is also taken into account. In the previous reports^24^, the capillary force in wettability fiber is exceeding 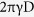 resulted from the stable solid/liquid contact line length between two knots. However, the high and low adhesion states present different fiber length, which results in the different force. For the d_20_ fiber, the 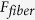 is insignificant related to 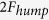. However, for the d_98_ fiber, the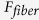 can not be ignored. In Fig. 3b, we give the water drop hanging on two knots in four conditions. In fact, the water drops is not dropped out with the time growing in U_h_ and D_h_ conditions. The water drops will creep down from the knot and further grow on the fiber. The 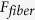 is big enough to balance the gravity of water drop. In conclusion, these investigations indicated that the capillary adhesion for a larger pearly hanging-drop on a rough-curve fiber could be controlled by the cooperation of three main forces: 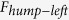, 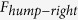 and 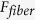. According to Fig. 3a,b and sketches in Fig. 3c, the most volume of hanging-drop could be predicted by different wetting condition.

Figure 4 depicts the water collection efficiency and the frequency appearing the ideal water drops during one hour under 95% humidity with four adhesion states, as H_h_, S_h_, U_h_ and D_h_. As for thinner fiber (d = 20 μm), spindles are L = 280 μm, H = 140 ± 8 μm and P = 720 μm. As for thicker fiber (d = 98 μm), spindles are L = 666 μm, H = 200 ± 13 μm and P = 720 μm, respectively. Both of the bio-inspired fiber is fabricated with 9% PCDF concentration. We calculated the water collection frequency (f) under different adhesion states during one hour. We define the maximum volume of 80% as the ideal drop state. During one hour, the frequency appearing ideal drops is one of measurement criterion on bioinspired fiber working stability. For different main fibers, the water collection efficiency is obviously distinct by the cooperation of 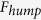 and 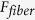. As the yellow and green dot shown in Fig. 4, on d_20_ fiber, the f is between 42% and 70%. While on d_98_ fiber, the f > 94%. It indicates that the less bioinspired fiber is more susceptible to the external environment, such as the surrounding water drops, humidity change and even air movement. The previous research^33^ have reported water collecting efficiency with different dip-coated mesh surface, and discussed the influence factors including wind velocity, coating of chemistry, water content. It can be seen that a number of influence factors should be considered to evaluate the water harvesting efficiency. Further, we next work will focus on the influence factors on water collecting efficiency.

This study is significant for developing smart materials to control liquid drop capture and controlled release by changing the adhesion condition. These findings are important in many potential applications, such as the transport of microdroplets^34^, water-harvesting from high humility^35^,^36^ and liquid collection in high efficiency^33^.

## Methods

### Fabrication of bioinspired fibers

The main (nylon) fibers with diameters (d) were 20 μm, 45 μm, 80 μm and 98 μm (defined as d_20,_ d_45,_ d_80_, and d_98_, respectively). The multi-scale humps were generated via Rayleigh instability technique^36^ and the dip-coating method. The main fibers were firstly immersed into poly(vinylidene fluoride, PVDF, M_w_ = 530000)/DMF (N,N-dimethylformamide, 9 wt%) solution. Then, the main fibers were vertically drawn out of the PVDF/DMF solution with a velocity 200 mm/min by a dip-coater machine (DipMaster-50, China). A coated polymer solution film quickly was broke up into periodic droplets and dried to resemble-spider-silk humps after 10 min in drying oven at 60 °C. Thus the bioinspired fibers were fabricated successfully. In detail of process, the dynamic wettability of formation on humps-on-strings was investigated by a using OCAmicro40 meter system (Data-Physics, Germany). And the size parameters, solid/liquid contact area and water hanging droplet volume were observed by analyst of software in Origin.

### Characterization of the structures

The structures of humps-on-strings were observed by scanning electron microscope (ESEM, Quanta FEG 250, EFI, America).

### Investigations on the water drop hanging under different adhesion situations

The experiments were carried on an OCAmicro40 meter system (Data-Physics, Germany) at room temperature. The fiber was carefully fixed on the sample frame. Numerous tiny water droplets were generated by an ultrasonic humidifier (YC-E350, Beijing YADU Science and Technology Co., Ltd.) as a fog flow and condensed on the fiber. The critical hanging-drops were recorded as drop hung on fiber with different orientations just the moment that the drops detached off via optical CCD camera in OCAmicro40 meter system.

## Additional Information

**How to cite this article**: Chen, Y. *et al*. Orientation-Induced Effects of Water Harvesting on Humps-on-Strings of Bioinspired Fibers. *Sci. Rep.* **6**, 19978; doi: 10.1038/srep19978 (2016).

## Supplementary Material

Supplementary Information

## Figures and Tables

**Figure 1:**
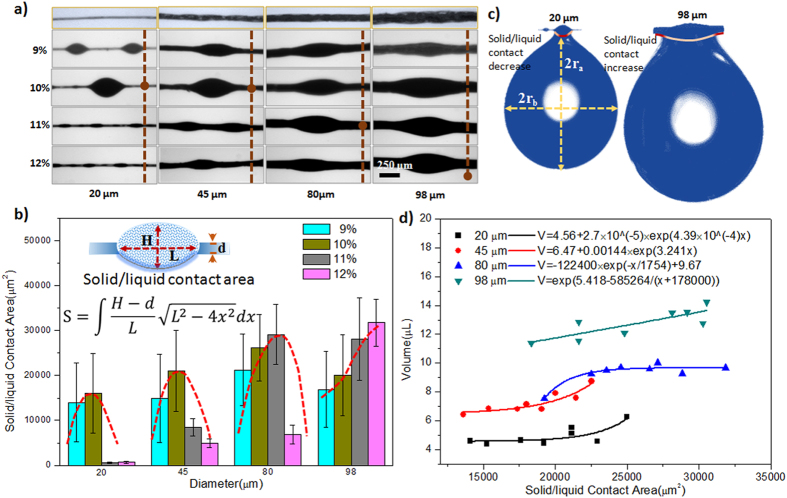
(**a**) Optical images of bioinspired fiber fabricated under different solution concentration and main fiber diameter. The concentration of PVDF/DMF solution can be 9%, 10%, 11% or 12% to be coated on the fibers of d = 20 μm, 45 μm, 80 μm and 98 μm, respectively. The red sphere between dotted lines reveals the parabola feature with the concentration increasing. (**b**) Relationship of solid/liquid contact area and fiber diameters and solution concentration of fabrication. The red dotted parabola lines depict an obvious tendency that solid/liquid contact surface will increase with the same trend of concentration until certain concentration value. (**c**) Compare of water droplet hanging on the solid/liquid interface area of single hump. The hang droplet is far larger on hump of d_98_ fiber than that on d_20_ fiber. The r_a_, r_b_ is the vertical and horizontal radius of droplet, respectively. (**d**) Statistical relationship of water droplet volume and solid/liquid contact area on d_20,_ d_45_, d_80_, d_98_ fibers. They are little linear. The volume of water droplet can be expressed as 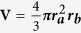. Generally, the tendency is that larger contact area induces larger hanging droplet in volume (e.g., d = 98 μm, V_max_ = 14 μL).

**Figure 2:**
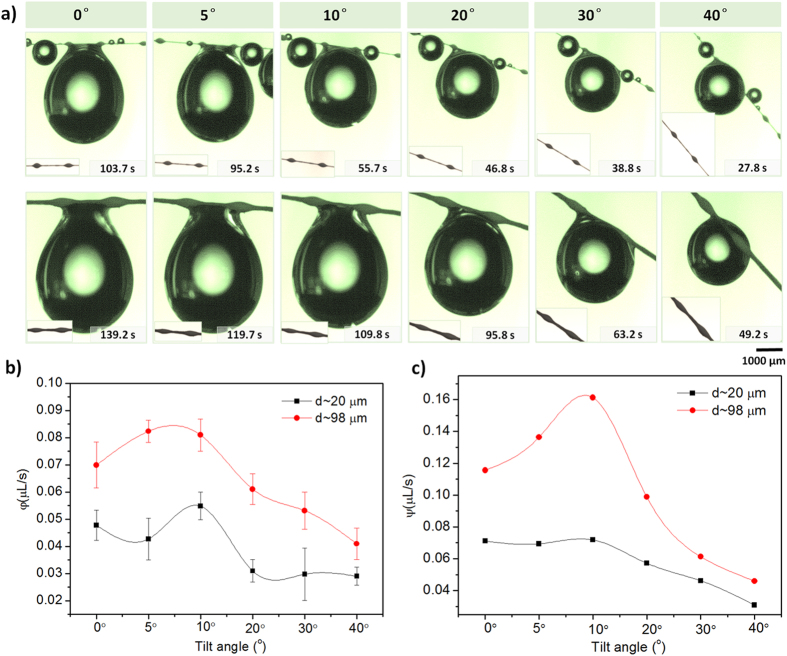
Water collecting efficiency on d_20_ and d_98_ fiber. (**a**) Optical images of humps hanging the most water drops with different tilt angles on two fibers. The bioinspired fibers are tilted to different angles (0°, 5°, 10°, 20°, 30°, 40°) under the humidity 95%. Generally, larger size fiber collects larger droplet (the bottom). The extreme volume of water drops are decreasing with the tilt angles increasing, and along with the decreasing time. (**b**) Water collection einmal efficiency Ψ under different tilt angles (0–40°). The most Ψ values on two hump-fibers are appearing a peak value between 5° and 10°. (**c**) Water collection efficiency Ψ (d = 20 μm, d = 98 μm) under different tilt angles (0–40°). There is the water drop collecting rate higher than one-way process (see • line, d = 98 μm).

**Figure 3:**
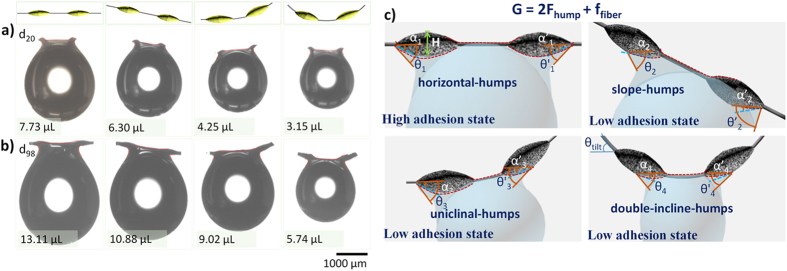
Optical images and scheme of four adhesion states. (**a**) Extreme volume of hanging water drops between two humps under four adhesion states on d_20_ fiber. The four adhesion states include horizontal-humps, slope-humps, uniclinal-humps and double-incline-humps. There are V = 7.73 μL, 6.30 μL, 4.25 μL and 3.15 μL, respectively. (**b**) Extreme volume of hanging water drops between two humps under four adhesion states on d_98_ fiber. There are V = 13.11 μL, 10.88 μL, 9.02 μL and 5.74 μL, respectively. (**c**) Illustration on the four adhesion situations for hanging water drops on bioinspired fiber. The capillary adhesion is described as: 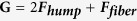, which is related to the apparent receding angle θ, and the semi-apex-angle of hump α. G is gravity of extreme hanging droplet. For the slope-humps and uniclinal-humps, both θ and α are asymmetrical on the left hump and right hump. Additionally, the solid/liquid contact lines (the red dotted line on the knots) are changing in four situations. It results in difference between 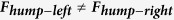 and 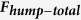 in four situations. Another force (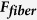) is also taken into account. For the smaller main fiber (d = 20 μm), the 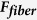 is insignificant related to 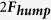. For the bigger main fiber (e.g., d = 98 μm), the 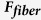 can not be ignored.

**Figure 4:**
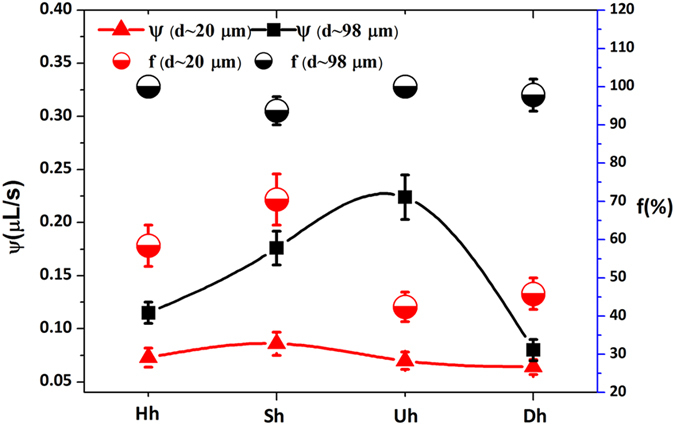
Compare on the water collection efficiency (Ψ) and the frequency (f) appearing the ideal water drops with four adhesion states for d_20_ and d_98_ fibers during one hour. The maximum volume of 80% is defined as the ideal situation of hanging-drop. For thinner fiber (d = 20 μm), f is between 42% and 70%, the Ψ can be 0.05–0.1 μL/s. For thicker one (d = 98 μm), there is f > 94%, Ψ can be up to 22–25 μL/s at maximum. H_h_, S_h_, U_h_, and D_h_ indicate four adhesion situations of horizontal-humps, slope-humps, uniclinal-humps and double-incline-humps, respectively. f is frequency in circles, Ψ is water collection efficiency in multi-circles.
