# Supplementary material for: Orientation-Induced Effects of Water Harvesting on Humps-on-Strings of Bioinspired Fibers
Source: Sci Rep. 2016 Jan 27;6:19978. doi: 10.1038/srep19978 (PMC4728488; doi:10.1038/srep19978)
Supplement: Supplementary Information [file srep19978-s1.pdf]

Supplementary Information

Orientation-Induced Effects of Water Harvesting on Humps-on-Strings of Bioinspired Fibers

Yuan Chen, Dan Li, Ting Wang, Yongmei Zheng*

Key Laboratory of Bio-Inspired Smart Interfacial Science and Technology of Ministry of Education, School of Chemistry and Environment, Beihang University, Beijing, 100191 (P. R. China).

***Corresponding author: e-mail: zhengym@buaa.edu.cn**

Content:

Supplementary Figures: Figure S1-S4

Supplementary Figure Legends: (Fig.S1-S4)

Figure S1

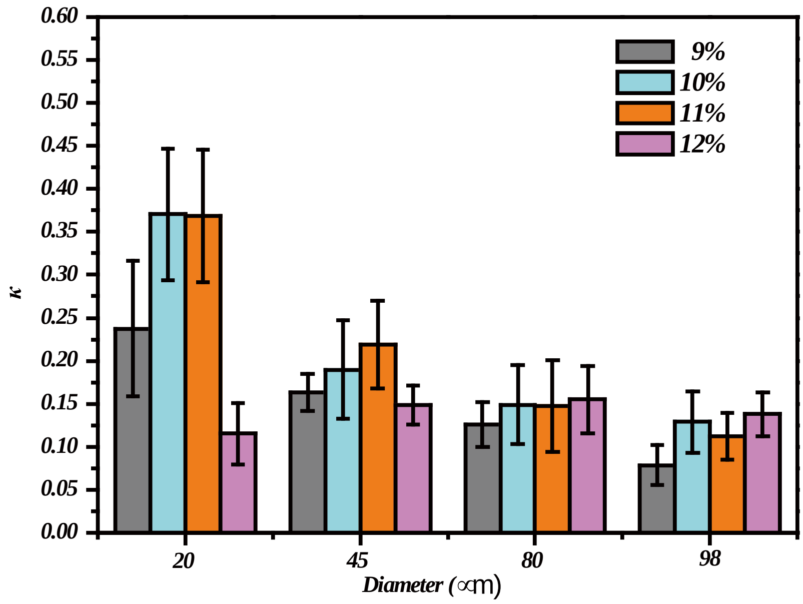

Figure S1. The κ value with concentration of PVDF (9%, 10%, 11% and 12%) and distinct main fiber diameters. We define $\kappa = \tan\alpha = (H - d)/L$, which α is the semi-axis angle. The κ value is measured with the joint-hump gradient, which directly affects the Laplace pressure difference. The average of κ value obviously decreases with the diameter increasing.

Figure S2

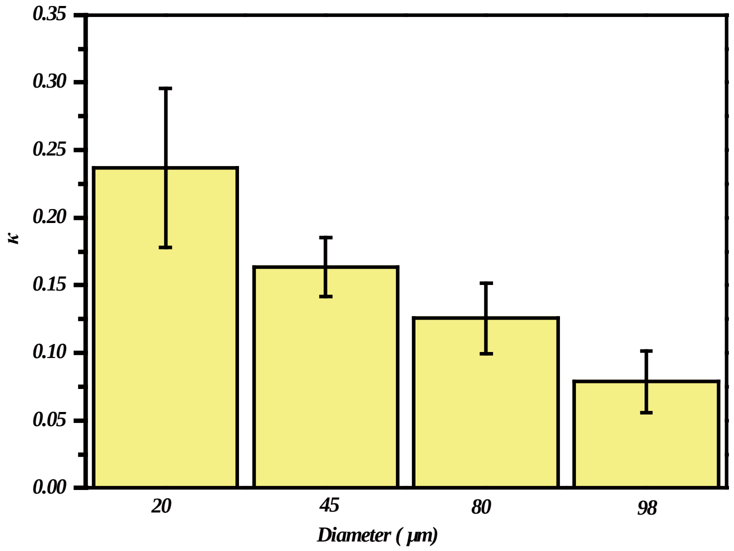

Figure S2. The κ value main fiber diameter increasing with 9% concentration of PVDF. The κ value decreases with the main fiber diameter increasing. The κ value is measured with the joint-hump gradient under different fabrication conditions.

Figure S3

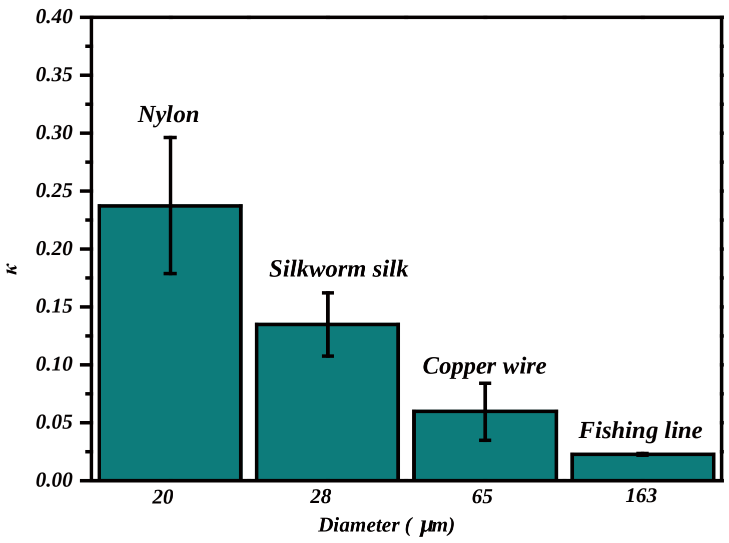

Figure S3. This κ value with different categories of fiber including nylon (diameter, 20 μm), silkworm silk (diameter, 23 μm), copper wire (diameter, 65 μm) and fishing line (diameter, 163 μm). The κ value is measured with the joint-hump gradient under different categories of main fibers.

Figure S4

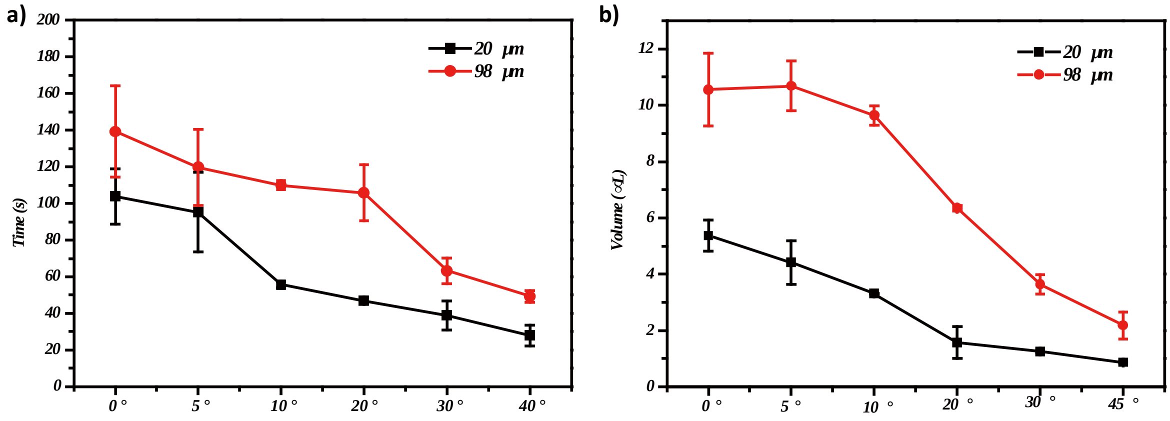

Figure S4. a) Bioinspired fibers tilt angles versus growing time of the most water drop volume. The first bioinspired fiber parameters include the main diameter of 20 μm, humps height of 146 μm, weight of 397 μm, κ=0.317. The second bioinspired fiber parameters include main diameter of 98 μm, humps height of 347 μm, weight of 1032 μm, κ=0.117. The collecting time of first bioinspired fiber is sharply reducing from ~103.7 s to ~27.8 s and the second one declines from ~139.2 s to ~49.2 s. b) Bioinspired fibers tilt angles versus the most growing water drop volumes.
